# ﻿Notes on *Carex* (Cyperaceae) from China (VIII): five new species and a new variety from southern and south-western China

**DOI:** 10.3897/phytokeys.188.77776

**Published:** 2022-01-07

**Authors:** Yi-Fei Lu, Xiao-Feng Jin

**Affiliations:** 1 School of Forestry and Bio-Technology, Zhejiang Agricultural and Forestry University, Lin’an, Zhejiang 311300, China Zhejiang Agricultural and Forestry University Hangzhou China; 2 College of Life Sciences, Zhejiang University, Hangzhou, Zhejiang, 310058, China Zhejiang University Hangzhou China

**Keywords:** *Carex*, China, Cyperaceae, new species, new variety, sedge, taxonomy

## Abstract

Our field surveys and specimen examination of *Carex* from southern to south-western regions in China resulted in the discovery of five new species and one new variety, which are here named as *Carexbrevihispida* X.F.Jin & Y.F.Lu (in sect. Surculosae), *C.puberuliutriculata* Y.F.Lu & X.F.Jin (sect. Clandestinae), *C.paratatsiensis* Y.F.Lu & X.F.Jin (sect. Aulocystis), *C.huanjiangensis* S.Yun Liang ex Y.F.Lu & X.F.Jin (sect. Decorae), *C.liangiana* X.F.Jin & Y.F.Lu and C.thibeticaFranch.var.angustifolia X.F.Jin & Y.F.Lu (sect. Rhomboidales).

## ﻿Introduction

*Carex* L. (Cyperaceae: tribe Cariceae), a morphologically diverse genus with about 2000 accepted species (Govaerts et al. 2021; Roalson et al. 2021), differs from the other genera within the family Cyperaceae by having unisexual flowers and a partially or completely enclosed prophyll, which is termed a utricle here. Most recent molecular phylogenetic studies of tribe Cariceae have demonstrated the genus *Carex* could be separated into four to six clades (Yen and Olmstead 2000; Roalson et al. 2001, 2021; Starr et al. 2008; Waterway et al. 2009, 2015; Jung and Choi 2013; Yano et al. 2014; Jiménez-Mejías et al. 2016; Martín-Bravo et al. 2019; Villaverde et al. 2020), in contrast to the earlier traditional classifications, based on morphology (Kükenthal 1909; Egorova 1999; Dai et al. 2000; Dai et al. 2010). As a result, *Kobresia* Willd., *Schoenoxiphium* Nees, *Cymophyllus* Mack. and *Uncinia* Pers. were merged into *Carex*, making the circumscription of *Carex* broader and equal to tribe Cariceae (Waterway et al. 2015; Roalson et al. 2021).

China is incredibly rich in species diversity of *Carex* and 527 recorded species are distributed from southern to northern regions and grow in various habitats, such as growing in forest, on grassland, in wetland or in sand (Dai et al. 2010). We carried out a taxonomic study of *Carex* from China since 2008. Recently, our field surveys and specimen examination of *Carex* have resulted in the discovery of several new taxa (Zhou and Jin 2014; Jin et al. 2015a, 2015b; Chen and Jin 2015; Lu and Jin 2017, 2018). In the present study, we describe another five distinctive new species and one new variety from the southern and south-western regions of China.

## ﻿Materials and methods

Over 20000 collections of *Carex* from East Asia, which were preserved in 27 Herbaria (alphabetically BM, CDBI, E, FJFC, FNU, GXMI, HGAS, HHBG, HNNU, HTC, HZU, IBK, IBSC, K, KUN, KYO, LBG, LE, N, NAS, P, PE, SZ, TI, WUK, ZJFC, and ZM), were examined. Our study is mainly based on these Herbarium collections and the descriptions for the new species and variety were also derived from the collected specimens. Width of leaves, length of glumes, utricles and nutlets were all measured from mature collections and descriptions of indumentum, colour of glumes, utricles and nutlets were observed from these specimens as well. These new taxa were critically compared with the type specimens of the relatives.

## ﻿Taxonomic treatment

### ﻿1. Carex brevihispida

Taxon classificationPlantaePoalesCyperaceae

X.F.Jin & Y.F.Lu sp. nov.

D19AC29B-4C2B-5D1D-AF63-F489DEB68927

urn:lsid:ipni.org:names:77235053-1

Figure 1A–G

#### Latin diagnosis.

*Haec species est affinis* C. kwangsiensi *F.T. Wang & Tang ex P.C. Li, a qua foliis caulinis, bracteis et pedunculis secundariis omnibus dense brevi-hispidis, squamis pistillatis 3–3.5 mm longis, dorso sparse pubescentibus vel subglabris differt*.

**Figure 1.**
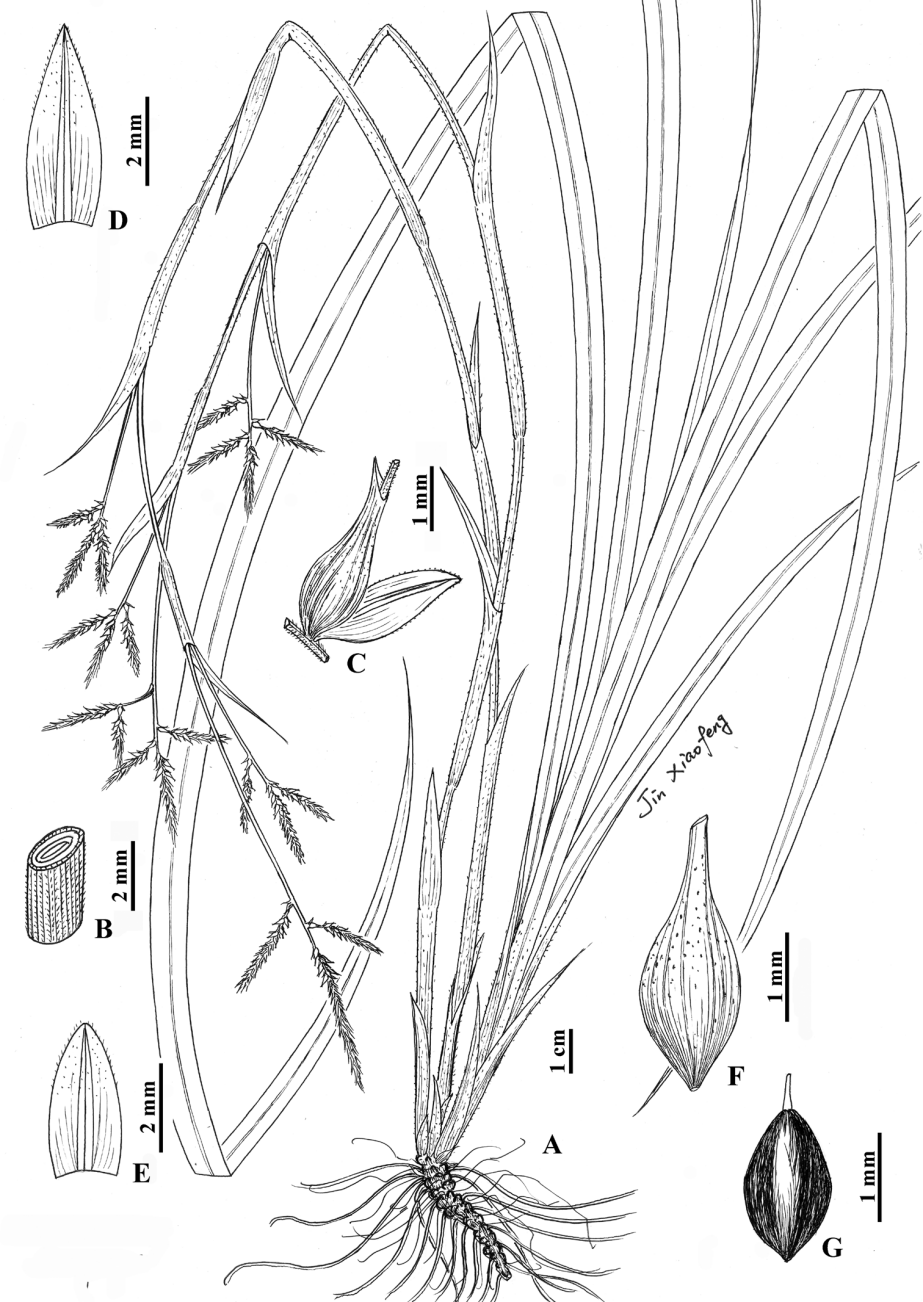
*Carexbrevihispida* sp. nov. **A** habit **B** part of culm **C** cladoprophyll **D** staminate glume **E** pistillate glume **F** utricle **G** nutlet (Drawn by Xiao-Feng Jin; based on holotype: *Xiao-Feng Jin et al. 4408* in ZM).

#### Type.

China. Guangxi: Baise, Youjiang Dist., Daleng, Mount Dawangling, 23°44'29.95"N, 106°23'28.85"E, by stream under forest, alt. 770 m, 26 Apr 2019, *X.F. Jin*, *W.J. Chen*, *X. Cai & Y.L. Xu 4408* (holotype: ZM; isotypes: ZJFC, ZM).

Rhizomes woody, thick, moniliform, creeping or obliquely ascending. Culms pseudo-lateral, 40–65 cm tall, ca. 2 mm thick, trigonous, base with grey-brown sheath. Leaves basal and cauline; basal leaves longer than or almost equal to culms, several ones forming a high shoot, blades flat or slightly revolute at margin, 5–7.5 mm wide, adaxially glabrous, abaxially glabrous or densely short-hispidulous along mid-ribs, scabrous on both surfaces and margins; cauline leaves spathe-like, lower ones rarely shortly leaf-like, purple-red when fresh, pale red-brown when dried, densely short-hispidulous. Bracts spathe-like, densely short-hispidulous. Panicle compound; inflorescence branches corymbose, single or binate, 3–5 cm long, 1.5–4.5 cm wide, with 2–5 spikes, rarely solitary; peduncles of inflorescence branches slender, densely short-hispidulous, exserted from bract sheath; inflorescence axes acutely trigonous, densely short-hispidulous; bractlets scale-like, lanceolate, 3–5 mm long, apex obtuse, glabrous, purple-red spotted; cladoprophylls utriculiform, ca. 2.5 mm long, distinctly thinly veined, orifice obliquely truncate. Spikes exserted from cladoprophylls, obliquely or horizontally patent, androgynous, 1–4.5 cm long, staminate part clavate or oblong, densely flowered, longer than or almost equal to pistillate part, pistillate part sparsely 1–8-flowered. Staminate glumes narrowly ovate-elliptic or elliptic-lanceolate, yellow-brown or brown, 3–4.5 mm long, apex acuminate or obtuse, upper margin ciliate, with yellow 3-veined costa. Pistillate glumes ovate or elliptic-ovate, yellow-brown, 3–3.5 mm long, apex obtuse, upper margin ciliate, abaxially sparsely pubescent or glabrous, with yellow 3-veined costa. Utricles brown-green, ovoid, obtusely trigonous, 2.5–3 mm long, obliquely patent, densely purple-red papillose, distinctly thinly veined, sparsely pubescent along veins, base with 0.2–0.3 mm long stipe, apex gradually contracted into a ca. 0.7 mm long beak, orifice obliquely truncate. Nutlets tightly enveloped, grey-brown, ovoid, trigonous, 1.7–2 mm long, base with ca. 0.3 mm long stipe; style base slightly thickened; stigmas 3.

#### Etymology.

The specific epithet ‘brevihispida’ refers to the culms, inflorescence bracts and peduncles of inflorescence branches that are all densely short-hispidulous.

#### Phenology.

Flowering and fruiting is from late March to late April.

#### Additional specimens examined.

China. Guangxi: Baise, Youjiang Dist., Daleng, Mount Dawangling, 23°44'29.95"N, 106°23'28.85"E, by stream under forest, alt. 770 m, 26 Apr 2019, *X.F. Jin*, *W.J. Chen*, *X. Cai & Y.L. Xu 4406* (ZM); the same locality, on cliff under forest, alt. 774 m, 26 Apr 2019, *X.F. Jin*, *W.J. Chen*, *X. Cai & Y.L. Xu 4399* (ZJFC, ZM).

#### Conservation status.

Near threatened (NT). The new species is currently known from the type locality, Mount Dawangling and grows by stream under forest. Tourists in the scenic region may interfere with the new species (IUCN 2019).

#### Notes.

The new species, *Carexbrevihispida*, has spathe-like cauline leaves and bracts and lateral culms, which morphologically belongs to sect. Surculosae in subg. Vigneastra (Dai et al. 2010). Recent phylogenetic hypotheses revealed the Siderostictae clade includes all species traditionally placed in sections *Siderostictae*, *Hemiscaposae* and *Surculosae* (Yano et al. 2014; Martín-Bravo et al. 2019; Villaverde et al. 2020; Roalson et al. 2021). It is morphologically similar to *Carexkwangsiensis*, but differs in having cauline leaves, bracts and secondary peduncles all densely short-hispidulous, pistillate glumes 3–3.5 mm long, dorsally sparsely pubescent or nearly glabrous. In *Carexkwangsiensis*, the cauline leaves, the bracts and the secondary peduncles are pubescent and the pistillate glumes are 2–2.5 mm long and dorsally pubescent.

### ﻿2. Carex puberuliutriculata

Taxon classificationPlantaePoalesCyperaceae

Y.F.Lu & X.F.Jin sp. nov.

236DC403-F0B8-5E78-B892-AA74F327C00C

urn:lsid:ipni.org:names:77235054-1

Figure 2A–E

#### Latin diagnosis.

*Affinis* C. pseudohumili *F.T. Wang & Y.L. Chang ex P.C. Li, a qua utriculis superne puberulis, apice pubescentibus, squamis pistillatis atro-purpureis vel brunneis manifeste longioribus, nucibus obovoideis recedit*.

**Figure 2.**
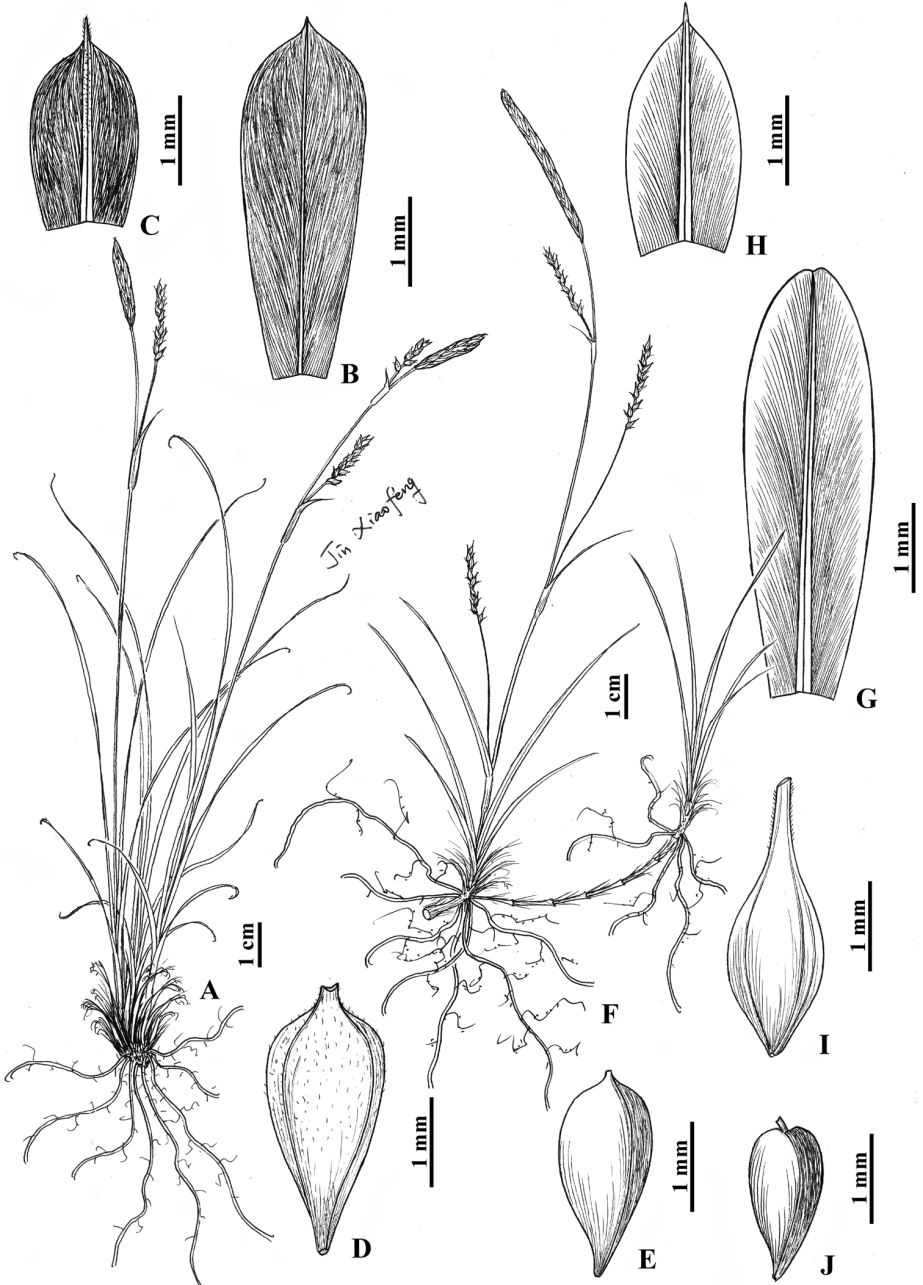
**A–E***Carexpuberuliutriculata* sp. nov. **A** habit **B** staminate glume **C** pistillate glume **D** utricle **E** nutlet **F–J***Carexparatatsiensis* sp. nov. **F** habit **G** staminate glume **H** pistillate glume **I** utricle **J** nutlet (Drawn by Xiao-Feng Jin; based on holotype: *X.H. Xiong 1129* for *C.puberuliutriculata* in ZM and holotype: *X.H. Xiong 999A* for *C.paratatsiensis* in ZM).

#### Type.

China. Sichuan: Baoxing, Fengtongzhai Natural Reserve, from Sandaoniupeng to Yuanyanyao, roadside grasses, alt. 3400–3700 m, 9 Jul 2017, *X.H. Xiong 1129* (holotype: ZM; isotypes: ZJFC, ZM).

Rhizomes short, woody. Culms central, caespitose, 6.5–20 cm tall, obtusely trigonous, smooth, base with yellow-brown or dark brown fibrous sheaths. Leaves almost equal to or shorter than culms; blades flat, 0.7–1.5 mm wide, margin slightly revolute, scabrous, apex curved or slightly circinate. Bracts spathe-like, shorter than inflorescence, base with 0.3–1.2 cm long sheaths. Spikes 2–4, upper ones aggregated, sometimes with lowest one exserted from basal culms; terminal spike staminate, oblong or clavate-cylindrical, 1.1–2 cm long, 1.5–3 mm wide, base with 0.8–1.2 cm long peduncles; lateral spikes pistillate, oblong or ovoid, 0.6–1 cm long, 3–3.5 mm wide, densely 7–15-flowered, peduncles erect, exserted from sheaths, 0.5–1.9 cm long. Staminate glumes obovate, purple-black or brown, 4–4.5 mm long, apex acute or obtuse, with pale yellow 1-veined pubescent costa excurrent into a mucro. Pistillate glumes ovate, purple-black or brown, 2.3–2.5 mm long, apex acute or obtuse, with yellow-brown 1- or 3-veined pubescent costa excurrent into a 0.5–1 mm long scabrous awn. Utricles pale yellow-brown, obovoid, obtusely trigonous, 3–3.3 mm long, membranous, obliquely patent, puberulent on upper part, laterally 2-veined, inconspicuously thinly veined, base cuneate and shortly curved stipitate, apex abruptly contracted into a ca. 0.3 mm long beak, orifice emarginate or obliquely truncate. Nutlets tightly enveloped, pale yellow, obovoid, trigonous, 2.2–2.3 mm long, base shortly stipitate, apex beakless; style base not thickened; stigmas 3.

#### Etymology.

The specific epithet ‘puberuliutriculata’ refers to the puberulent utricles of the new species.

#### Phenology.

Flowering and fruiting is in early July.

#### Additional specimen examined.

China. Sichuan: Baoxing, Fengtongzhai Natural Reserve, from Sandaoniupeng to Yuanyanyao, in grasses, alt. 3400–3700 m, 9 Jul 2017, *X.H. Xiong 1128* (ZJFC, ZM).

#### Conservation status.

Least Concern (LC). The new species is a common grass in the meadow of Fengtongzhai at an elevation from 3300 to 4000 m. Local animal grazing may have an impact on this species (IUCN 2019).

#### Notes.

The new species is somewhat morphologically similar to *Carexpseudohumilis* in having hairy utricles, inconspicuous beaks and leaves curved or slightly circinate at the apex (Dai et al. 2000, 2010), but it differs from the latter in having pistillate glumes purple-black or brown, utricles puberulent on upper part and longer than pistillate glumes and nutlets obovoid. Herein, it is placed in sect. Clandestinae, which is part of the poorly resolved Hallerianae-Digitatae clade (Roalson et al. 2021).

### ﻿3. Carex paratatsiensis

Taxon classificationPlantaePoalesCyperaceae

Y.F.Lu & X.F.Jin sp. nov.

8DCAB89C-C410-59C2-9B94-281DA6FE734F

urn:lsid:ipni.org:names:77235056-1

Figure 2F–J

#### Latin diagnosis.

*Haec species nova* C. tastiensi *(Franch.) Kük. affinis est, sed utriculis brevioribus, 3*–*3.2 mm longis, membranaceis, squamis pistillatis ovatis vel late ovatis, nucibus obovoideis, stigmatibus 2 vel 3 differt*.

#### Type.

China. Tibet: Mêdog, Dayandong, 29°25'45.54"N, 95°02'58.37"E, in thickets on slope, alt. 2950 m, 7 Jun 2017, *X.H. Xiong 999A* (holotype: ZM; isotypes: ZJFC, ZM).

Rhizomes slender, long, woody, long-stoloniferous. Culms central, 14–30 cm tall, slender, obtusely trigonous, lower part smooth and upper part scabrous, base with red-brown fibrous sheaths. Leaves shorter than culms; blades flat, 1–2 mm wide, margin scabrous. Bracts shortly leaf-like or uppermost setaceous, shorter to longer than inflorescence, base with 0.5–2 cm long sheaths. Spikes 2–4, remote; terminal 1 or 2 spikes staminate, narrowly cylindrical, 1–3.5 cm long, 1–2.5 mm wide, base with 0.3–6 cm long peduncles; lateral spikes pistillate, single or rarely binate, cylindrical, 0.8–2.7 cm long, 2.5–4 mm wide, 8–18-flowered, peduncles erect, slender, 0.3–7.5 cm long, exserted from sheaths. Staminate glumes obovate-lanceolate, red-brown, 5–5.5 mm long, apex acute or emarginate, with yellow 3-veined costa excurrent into a mucro. Pistillate glumes ovate or broadly ovate, red-brown, 2.5–2.8 mm long, margin whitish hyaline, apex acute or emarginate, with yellow-brown 3-veined costa excurrent into a mucro. Utricles red-brown and yellow-green below, ellipsoid, obtusely trigonous, 3–3.2 mm long, membranous, obliquely patent, inconspicuously several thinly veined, base cuneate and shortly stipitate, apex gradually contracted into a ca. 1 mm long beak, orifice truncate or 2-lobed with minute teeth, margin barbate. Nutlets tightly enveloped, yellow, obovoid, trigonous, 1.8–2 mm long, apex with ca. 0.3 mm long curved beak; style base not thickened; stigmas 2 or 3.

#### Etymology.

The specific epithet ‘paratatsiensis’ refers to the similarity with *Carextatsiensis*.

#### Phenology.

Flowering and fruiting is in early June.

#### Additional specimen examined.

China. Tibet: Mêdog, Dayandong, 29°25'45.54"N, 95°02'58.37"E, in thickets on slope, alt. 2950 m, 7 Jun 2017, *X.H. Xiong 999B*, *999C* (ZJFC, ZM).

#### Conservation status.

Data Deficient (DD). There is inadequate information for distribution and population status and we could not make a direct assessment of its risk of extinction now (IUCN 2019).

#### Notes.

This new species is similar to *Carextatsiensis*, which was placed in sect. Aulocystis, but differs in having utricles shorter (3–3.2 mm long), membranous, pistillate glumes ovate or broadly ovate and nutlets obovoid with 2 or 3 stigmas. Based on the phylogenetic hypotheses, the traditional taxonomic section Aulocystis was polyphyletic and clustered with some species of sect. Clandestinae, which made it a heterogeneous group (Roalson et al. 2021). In some descriptions of *Carextatsiensis* (Dai et al. 2000, 2010), the utricles were described as membranous, but our examination showed the utricles of *C.tatsiensis* are thin-coriaceous or coriaceous (Jin and Lu 2020).

### ﻿4. Carex huanjiangensis

Taxon classificationPlantaePoalesCyperaceae

S.Yun Liang ex Y.F.Lu & X.F.Jin sp. nov.

CC1853F9-DB2C-5389-8DDE-35C6A0893F05

urn:lsid:ipni.org:names:77235058-1

Figure 3A–H

#### Latin diagnosis.

*Haec species* C. perakensi *C.B. Clarke affinis est, sed squamis staminatis et pistillatis fulvis, utriculis 9*–*10 mm longis, glabris, rostris margine serrulatis, stylis basi glabris differt*.

**Figure 3.**
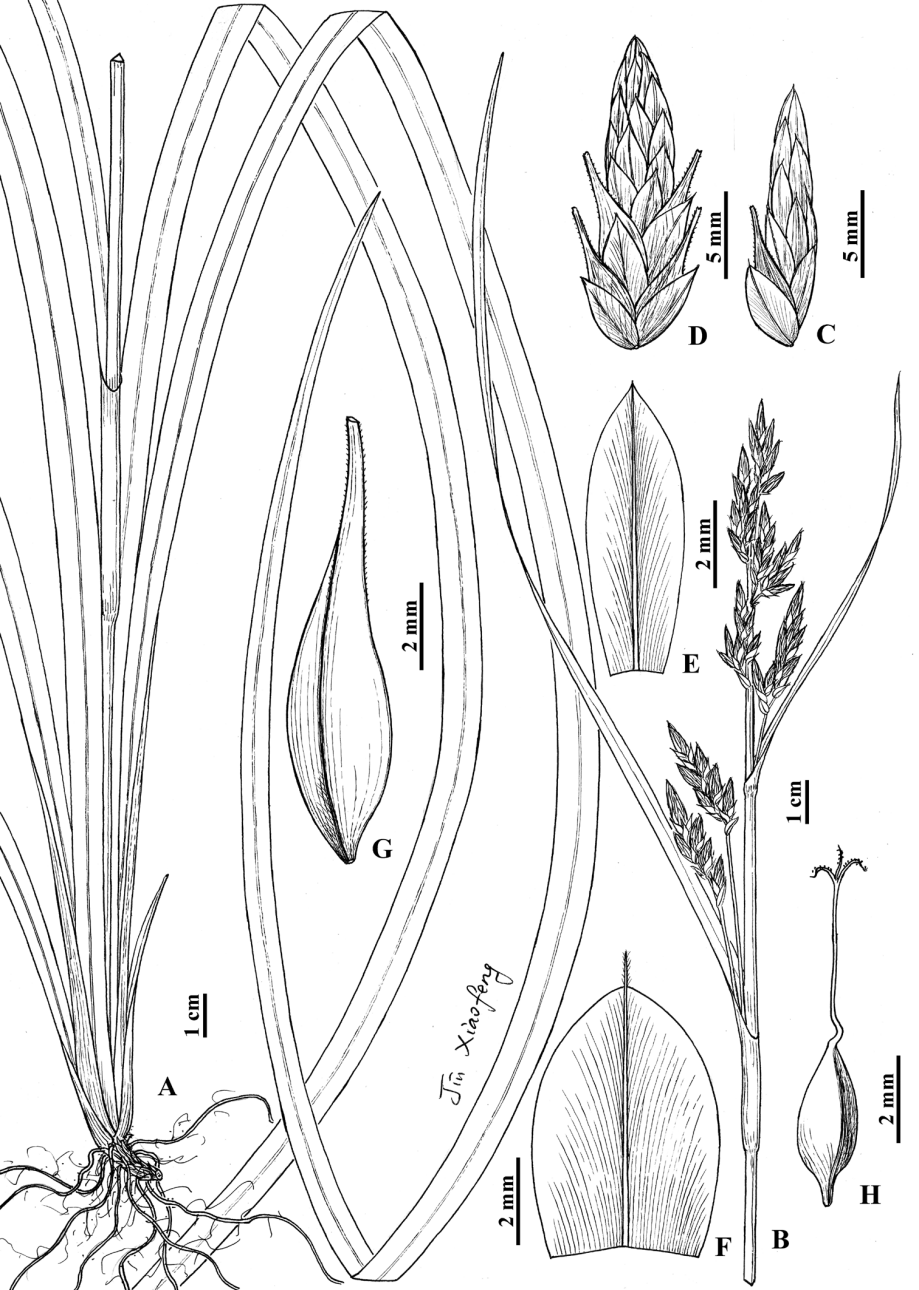
*Carexhuanjiangensis* sp. nov. **A** lower part of habit **B** upper part of habit **C** lateral spike **D** terminal spike **E** staminate glume **F** pistillate glume **G** utricle **H** nutlet (Drawn by Xiao-Feng Jin; based on holotype: *Beijing Exped 894059* in PE).

#### Type.

China. Guangxi: Huanjiang, Dongxing Town, Jiupengtun, by stream, alt. 1200 m, 22 May 1989, *Beijing Exped 894059* (holotype: PE).

Rhizomes dark brown, woody, stiff, sometimes stoloniferous, with black-brown fibrous roots. Culms central, loosely caespitose, 40–80 cm tall, trigonous. Leaves basal and cauline; basal leaves longer than or almost equal to culms, blades 5–12 mm wide, coriaceous, apex acuminate, margin scabrous; cauline leaves 1 or 2, sometimes absent, slightly longer than inflorescence, blades 5–9 mm wide, coriaceous, apex acuminate, margin scabrous. Bracts leaf-like, longer than or slightly longer than inflorescence, 2.5–8 mm wide, with the lowest sheath to 2 cm long, upper sheaths shorter or sheath absent. Panicle compound, 15–28 cm long, 2 inflorescence branches in each bract sheath, rarely single; inflorescence branch 5–8.5 cm long, base pedunculate; bractlets scale-like, broadly ovate-round, yellow-brown, 4–5 mm long, ca. 4.5 mm wide, apex obtuse or mucronate, with yellow 1-veined costa. Spikes 13–46, sessile, 4–7 in a racemose; terminal spikes narrowly ovate or ovate-elliptic, 13–17 mm long, 6–7 mm wide, base with 3–6 pistillate flowers, staminate part 6–8 mm long; lateral spikes elliptic-lanceolate, 7–12 mm long, 2–3.5 mm wide, base with single pistillate flower, staminate part 5–8 mm long. Staminate glumes narrowly obovate or obovate-elliptic, yellow-brown, 6.5–7 mm long, apex acuminate or obtuse, with yellow 1-veined costa. Pistillate glumes broadly ovate, yellow-brown, 6.5–7 mm long, apex obtuse, with yellow 1-veined costa excurrent into a 0.5–1 mm long scabrous awn. Utricles pale brown, obovoid, compressed trigonous, 9–10 mm long, obliquely patent, glabrous, abaxially, and adaxially with 9 or 10 veins, respectively, apex gradually contracted into a 3–3.5 mm long erect beak, orifice obliquely truncate, margin barbate. Nutlets loosely enveloped, grey-brown, narrowly ovoid, trigonous, ca. 3.5 mm long, base with ca. 0.7 mm long stipe; style base curved, not thickened; stigmas 3.

#### Etymology.

The specific epithet ‘huanjiangensis’ refers to the type locality of this new species, Huanjiang County of Guangxi Zhuang Autonomous Region.

#### Phenology.

Flowering and fruiting is in early to late May.

#### Additional specimens examined.

China. Guangxi: Huanjiang, Jiuren Forestry Farm, on slope under forest, alt. 1450 m, 21 May 1989, *Beijing Exped. 892928* (PE); the same locality, alt. 980 m, 21 May 1989, *Beijing Exped. 893056* (PE); on slope under forest, alt. 1000 m, 25 May 1989, *Beijing Exped. 895076* (PE); by stream, alt. 700 m, 25 May 1989, *Beijing Exped. 895011* (PE); Rongshui, Xiangcaopeng, by stream, alt. 1000 m, 2 May 1989, *Beijing Exped. 892115* (PE).

#### Conservation status.

Least Concern (LC). The new species grows on slopes under the forests in Huangjiang and Rongshui Counties, where are seriously disturbed by local people (IUCN 2019).

#### Notes.

Based on phylogenetic analyses, the large Decora clade includes most species in sections *Decorae* and *Indicae* (Roalson et al. 2021). Herein, we identified the former section as lacking utriculiform cladoprophylls, whereas they are present in section Indicae. This new species is similar to *Carexperakensis*, but differs in having both staminate and pistillate glumes yellow-brown, utricles longer (9–10 mm long), glabrous, with beak margins barbate and styles glabrous. In *Carexperakensis*, the glumes are pale yellow-brown, the utricles are 4.5–6 mm long, densely hispidulous and styles are sparsely barbate.

### ﻿5. Carex liangiana

Taxon classificationPlantaePoalesCyperaceae

X.F.Jin & Y.F.Lu sp. nov.

DFA4E4C9-C57F-5F03-A142-0A784B3D254B

urn:lsid:ipni.org:names:77235060-1

Figure 4A–E

#### Latin diagnosis.

*Species nova est affinis* C. diplodo *Nelmes, a qua spicis terminalibus androgynis, squamis pistillatis ovatis, apice breviter et scabrose aristatis, utriculis glabris, nucibus apice erostris facile differt*.

**Figure 4.**
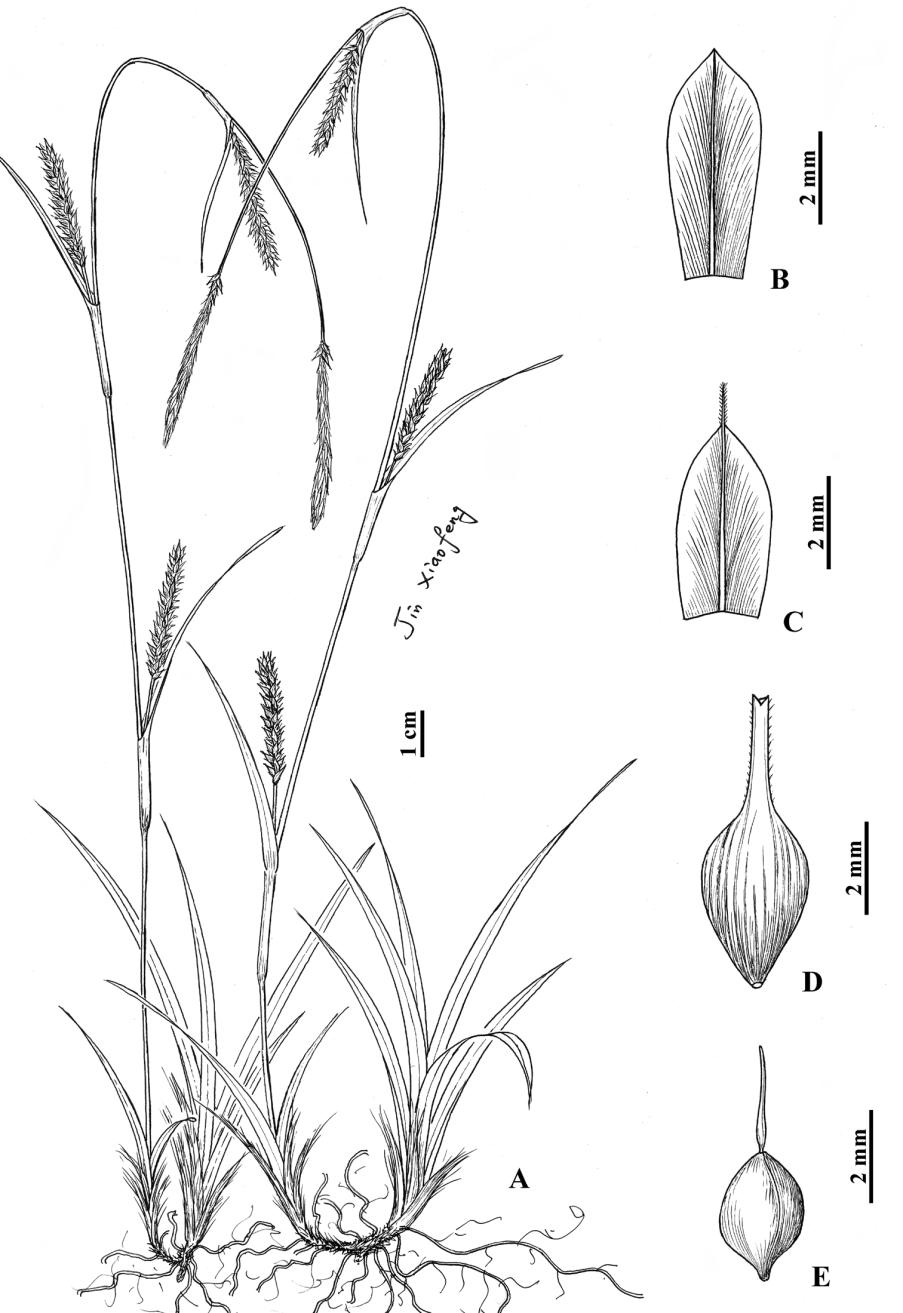
*Carexliangiana* sp. nov. **A** habit **B** staminate glume **C** pistillate glume **D** utricle **E** nutlet (Drawn by Xiao-Feng Jin; based on holotype: *X.F. Jin*, *Y.F. Lu & X.H. Xiong 4518* in ZM).

#### Type.

China. Sichuan: Kangding, Zhonggucun, Wachang, 30°15'16.72"N, 101°52'47.75"E, in grass along stream, alt. 3061 m, 1 Aug 2019, *X.F. Jin*, *Y.F. Lu & X.H. Xiong 4518* (holotype: ZM; isotypes: ZJFC, ZM)

Rhizomes creeping, woody, stiff, with many brown fibrous roots. Culms central, loosely caespitose, 25–60 cm tall, acutely trigonous, smooth, glabrous, with single bract-like leaf near base. Leaves basal, far shorter than culms; blades less than 12 cm long, flat, 2.5–5.5 mm wide, coriaceous, apex acuminate, margin slightly scabrous. Lower bracts shortly leaf-like, longer than spikes, upper ones setaceous, shorter than spikes, all bracts sheathed; sheaths 0.5–2.5 cm long. Spikes 4 or 5 in a racemose, terminal spike androgynous, clavate-cylindrical, 1.5–3 cm long, 2–2.5 mm wide, base with 1–6 pistillate flowers and a 1–2.5 cm long peduncle, lateral spikes pistillate, sometimes with 3–6 staminate flowers at apex, cylindrical, 1–2.5 cm long, 5–5.5 mm wide, 8–24-flowered, lower 1 or 2 peduncles exserted from sheaths, upper ones enclosed. Staminate glumes obovate, yellow-brown, 4.5–5 mm long, apex acute, with yellow 1-veined costa excurrent into a mucro. Pistillate glumes ovate, yellow-brown, margin whitish hyaline, 4–4.3 mm long, apex acute or obtuse, with yellow 1-veined costa excurrent into a 0.5–1 mm scabrous awn. Utricles yellow-brown, ovoid, obtusely trigonous, 5–6 mm long, obliquely patent, abaxially thinly 11–14-veined, adaxially thinly 8- or 9-veined, glabrous, apex gradually contracted into a 2–2.5 mm long beak, orifice 2-lobed with short teeth, margin barbate or smooth. Nutlets tightly enveloped, yellow-brown, broadly ovoid, trigonous, 2.3–2.5 mm long, base with a stipe ca. 0.3 mm long; style base slightly thickened; stigmas 3.

#### Etymology.

The specific epithet ‘liangiana’ is in honour of Prof. Song-Yun Liang, who is a Chinese researcher on the taxonomy of Cyperaceae and Liliaceae.

#### Phyeology.

Flowering and fruiting is from late June to early August.

#### Additional specimens examined.

China. Sichuan: Kangding, Zhonggucun, Wachang, 30°15'16.72"N, 101°52'47.75"E, in grass along stream, alt. 3061 m, 1 Aug 2019, *X.F. Jin*, *Y.F. Lu & X.H. Xiong 4512* (ZJFC, ZM); the same locality, in grass, alt. 3370 m, 29 Jul 1963, *W Sichuan Exped. (K.C. Kuan & W.T. Wang) 505* (PE). Gansu: Zhouqu, Wuping Dist., Shatan Forestry Farm, in wetland, alt. 2300 m, 26 Jun 1964, *P.C. Kuo 5115* (WUK).

#### Conservation status.

Vulnerable, VU B2aC1 (IUCN 2019). This new species is known from two localities, Kangding of Sichuan Province and Zhouqu of Gansu Province, the area of occupancy is less than 10000 km^2^ and the estimated individuals are less than 5000 individuals in the two populations.

#### Notes.

It is a remarkable species in sect. Rhomboidales with the terminal spikes androgynous and the nutlets not beaked at the apex. It is somewhat similar to *Carexdiplodon* in the shape of the utricles and nutlets, but the new species has culms that are loosely tufted with elongate rhizomes and the nutlets neither concave nor excavated on the faces, which differentiates it from the majority of species in sect. Rhomboidales (Jin and Zheng 2013; Jin et al. 2014). Further phylogenetic study is needed to establish its relationships.

### ﻿6. Carex thibetica Franch. var. angustifolia

Taxon classificationPlantaePoalesCyperaceae

X.F.Jin & Y.F.Lu var. nov.

1061AFED-06EA-5AAC-B2E6-F4C3F3F102E6

urn:lsid:ipni.org:names:77235062-1

Figure 5A–E

#### Latin diagnosis.

*A var.*thibetica*differt foliis 4–6.5 mm latis, spicis staminatis 1.5–2 mm latis, utriculis longioribus 9–12 mm longis*.

**Figure 5.**
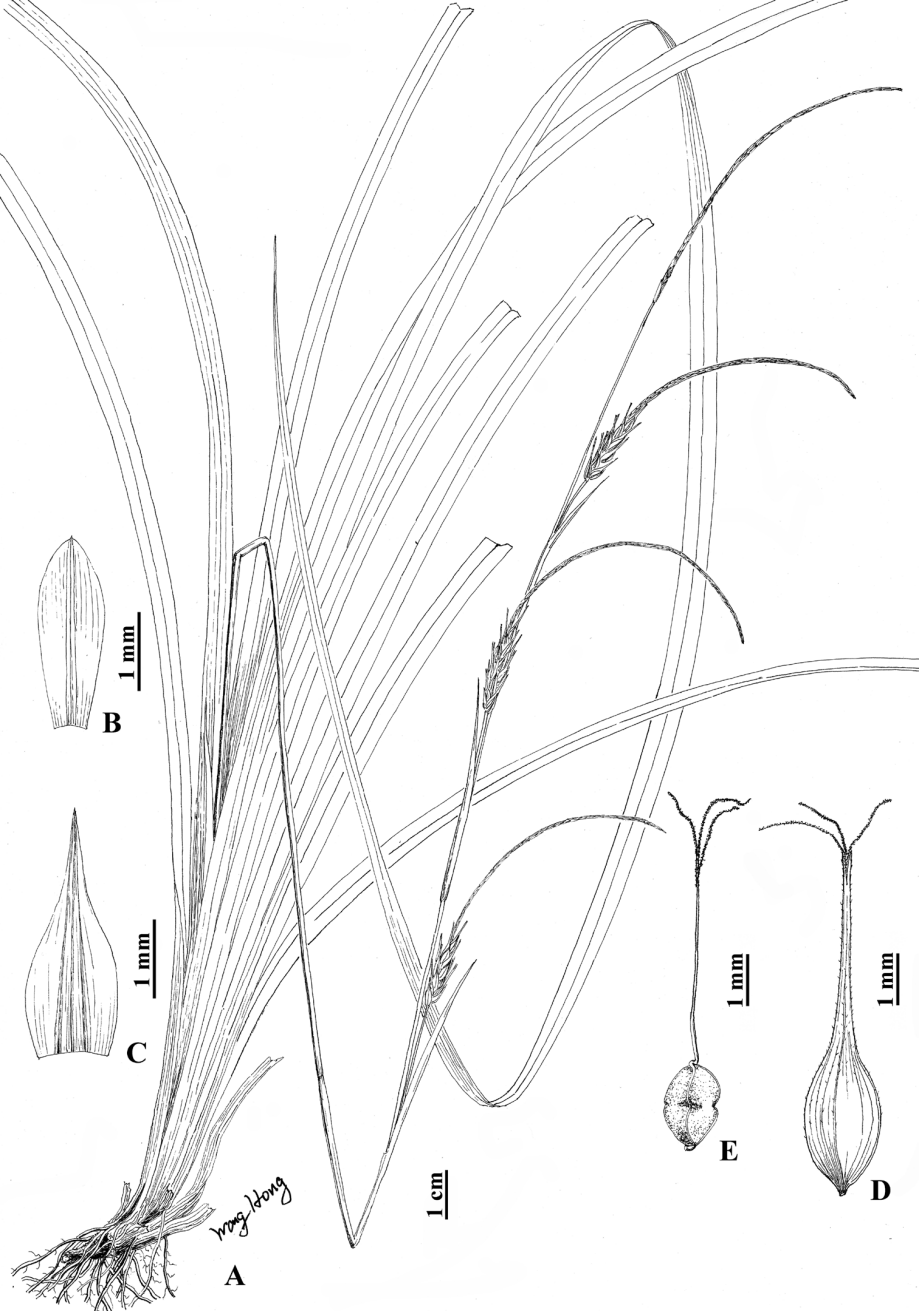
Carexthibaticavar.angustifolia var. nov. **A** habit **B** staminate glume **C** pistillate glume **D** utricle **E** nutlet (Drawn by Hong Wang; based on holotype: *H. Wang 1434* in ZM).

#### Type.

China. Hunan: Dongan, Shunhuangshan Forestry Park, Butterfly Valley, by stream under forest, alt. 680 m, 21 May 2017, *H. Wang 1434* (holotype: ZM; isotypes: ZJFC, ZM).

Rhizomes short or elongate, woody, stiff, with many brown fibrous roots. Culms lateral, loosely caespitose, 20–55 cm tall, obtusely trigonous, smooth, glabrous. Leaves basal, slightly shorter to longer than culms; blades flat, 4–6.5 mm wide, coriaceous, apex acuminate, lower surfaces and margin slightly scabrous. Bracts shortly leaf-like, shorter than spikes, sheathed; sheaths 1.5–2.5 cm long. Spikes 3 or 4 in a racemose; terminal spike staminate, narrowly linear-cylindrical, 3.5–8 cm long, 1.5–2 mm wide, base with 4–8 cm long peduncles; lateral spikes androgynous, staminate part longer than pistillate part, cylindrical, 4–9 cm long, 7–10 mm wide, 8–20-flowered (pistillate), with peduncles exserted from sheaths. Staminate glumes lanceolate or lanceolate-oblong, pale yellow-green, 7–7.5 mm long, apex acuminate, with pale brown 1-veined costa. Pistillate glumes narrowly ovate, pale green-brown or brown, 8.5–11 mm long, apex acuminate, with green or brown-green 3-veined costa. Utricles yellow-brown or brown, rhombic-ovoid, obtusely trigonous, 9–12 mm long, slightly longer than pistillate glumes, nearly erect or obliquely patent, distinctly thinly veined, sparsely puberulent on upper part, apex abruptly contracted into a 4.5–6 mm long beak, orifice 2-lobed with long teeth, margin barbate. Nutlets tightly enveloped, brown, rhombic-ovoid, trigonous, 3–4 mm long, base with a ca. 0.5 mm long curved stipe, apex abruptly contracted into a coiled short beak, with 3 angles constricted at middle and sides concave above and below; style base slightly thickened; stigmas 3.

#### Etymology.

The variety epithet ‘angustifolia’ refers to the narrower leaves (4–6.5 mm wide) than the typical variety (6–17 mm wide).

#### Phenology.

Flowering and fruiting is from mid-April to late May.

#### Additional specimens examined.

China. Hunan: Dongan, Shunhuangshan Forestry Park, Butterfly Valley, by stream under forest, alt. 680 m, 21 May 2017, *H. Wang 1433* (ZJFC, ZM); the same locality, alt. 730 m, 19 Apr 2018, *W.J. Chen 2394* (HTC, ZM), *2395* (ZJFC, ZM), *2396* (ZJFC, ZM). Suining, Huangsang Natural Reserve, Quyougu, under forest, alt. 755 m, 26 Apr 2014, *J.J. Zhou & Z.P. Song 1404223* (CSFI); the same locality, Banchong, under forest, alt. 906 m, 2 May 2014, *J.J. Zhou & Z.P. Song 1405022* (CSFI).

#### Conservation status.

Least Concern (LC). The new variety is known from four localities in southern Hunan Province, but two of them are seriously disturbed by local people (IUCN 2019).

#### Notes.

The new variety differs from the typical variety in having the leaves narrower, 4–6.5 mm in width, terminal staminate spikes 1.5–2 mm in width and utricles longer, 9–12 mm long. It differs from Carexthibaticavar.pauciflora in having lateral spikes with densely 8–20 pistillate flowers and utricles longer, 9–12 mm long.

## Supplementary Material

XML Treatment for Carex brevihispida

XML Treatment for Carex puberuliutriculata

XML Treatment for Carex paratatsiensis

XML Treatment for Carex huanjiangensis

XML Treatment for Carex liangiana

XML Treatment for Carex thibetica Franch. var. angustifolia
